# Establishment of Multiplex Digital PCR Assay for Detection of Four Porcine Enteric Coronaviruses

**DOI:** 10.3390/ijms26178731

**Published:** 2025-09-08

**Authors:** Xiao Han, Kexin Chen, Hui Qiu, Pengli Kong, Xiaoliang Li, Linglin Fu, Huan Li, Jinru Zhou, Xiaofeng Zhang, Jiangbing Shuai

**Affiliations:** 1 Zhejiang Academy of Science and Technology for Inspection and Quarantine, Hangzhou 310016, China; hanx@zju.edu.cn (X.H.); ksxx9954@stu.zafu.edu.cn (K.C.); qh@zaiq.org.cn (H.Q.); kongpengli1110@163.com (P.K.); 2 Department of Veterinary Medicine, College of Animal Sciences, Zhejiang University, Hangzhou 310058, China; xlli@zju.edu.cn; 3 Food Safety Key Laboratory of Zhejiang Province, School of Food Science and Biotechnology, Zhejiang Gongshang University, Hangzhou 310018, China; fulinglin@mail.zjgsu.edu.cn (L.F.); huanli@mail.zjgsu.edu.cn (H.L.); zhoujinru33@163.com (J.Z.)

**Keywords:** SADS-CoV, PEDV, PDCoV, TGEV, dPCR, porcine enteric coronavirus

## Abstract

Porcine enteric coronaviruses (CoVs), including swine acute diarrhea syndrome coronavirus (SADS-CoV), porcine epidemic diarrhea virus (PEDV), porcine deltacoronavirus (PDCoV), and porcine transmissible gastroenteritis virus (TGEV), are major pathogens causing porcine viral diarrhea syndrome (VDS), which brings significant economic losses to the swine industry; distinguishing between these clinically similar viruses has become a serious challenge. We developed a highly specific and interference-resistant porcine CoV multiplex digital PCR (dPCR) assay. The assay exhibited robust anti-interference capabilities, as the concentrations of the four viruses did not affect their accurate quantification. The coefficients of variation (CV%) of intra-batch and inter-batch repeatability for all target viruses were less than 11%. The limit of quantification (LoQ) of this dPCR assay reached 7.5 copies/reaction for each target, and it was one order of magnitude more sensitive than qPCR. The limits of detection (LoD) for SADS-CoV, PEDV, PDCoV, and TGEV were 2.72, 3.00, 3.56, and 3.19 copies/reaction, respectively. A total of 408 known samples were used for validation tests, and the results were highly consistent with the known conditions, showing a compliance rate of 97–100%. The diagnostic specificity (Dsp) of the method was 99–100%. In conclusion, the developed multiplex dPCR assay is highly suitable for early detection and quarantine in four porcine CoVs. The results indicate that this dPCR method is characterized by high specificity, anti-interference capabilities, repeatability, and high sensitivity. It also demonstrates a high compliance rate and diagnostic specificity in sample detection. This multiplex dPCR will contribute to the control of porcine enteric CoV-caused VDS and provide clues for subsequent research.

## 1. Introduction

Coronaviruses (CoVs) are single-stranded, positive-sense RNA viruses with capsids. Their surface spike proteins interact with cell receptors in susceptible animals, facilitating invasion [1,2]. They are the largest known RNA viruses, capable of infecting a diverse range of vertebrates [3]. In the swine industry, porcine coronaviruses are major pathogens causing viral diarrhea, leading to significant economic losses and challenges in swine farming. Common porcine enteric coronaviruses include swine acute diarrhea syndrome coronavirus (SADS-CoV), porcine epidemic diarrhea virus (PEDV), porcine deltacoronavirus (PDCoV), and porcine transmissible gastroenteritis virus (TGEV). SADS-CoV is a new coronavirus discovered in Guangdong, China in recent years, belonging to the genus *α*-coronavirus, featuring a typical coronavirus structure and a genome length of approximately 27 kb [4,5]. It causes acute diarrhea and acute vomiting in infected piglets and a rapid decline in the body mass of newborn piglets within 5 days of age, leading to acute death, with a lethality rate of more than 90% [6]. PEDV, also an α-coronavirus, is a non-segmented, positive-sense, single-stranded RNA virus [7]. It infects pigs of all ages, particularly neonatal piglets, with a high incidence rate of more than 90%, and the incidence rate in sows ranges from 20 to 90% [8,9,10]. PDCoV, a *δ*-coronavirus, induces severe intestinal lesions in neonatal piglets [11,12]. In studies, the lumen of the small intestine in the infected piglets accumulated large amounts of yellowish fluid, the intestinal wall was thin and transparent, the intestinal villi of the jejunum and the ileum were severely atrophic, and enterocytes showed mild cytoplasmic vacuolization [4,11], with symptoms such as vomiting, acute diarrhea, dehydration and even death in severe cases [13]. TGEV, another *α*-coronavirus, targets porcine intestinal epithelial cells via the fecal–oral route and spreads rapidly, affecting all age groups [14,15,16]. Usually, in piglets within 20 d of age, if infected with TGEV, the survival time is less than 4 d [16]. The clinical characteristics of the onset of the disease are lethargy, depression, a short period of rapid weight loss, severe watery diarrhea, vomiting, and dehydration; the mortality rate can be up to 100% [17].

Since 2023, SADS-CoV outbreaks have mostly occurred in Southern China and have affected Guangdong, as well as its adjacent provinces, Fujian, Guangxi, and Jiangxi [18,19,20]. Although SADS-CoV has not reached pandemic levels, its novel structural properties and strong cell invasion capabilities pose a risk of zoonotic evolution, warranting heightened quarantine and control measures [21]. PEDV was first identified in the United Kingdom in 1971 [22] and began spreading in Asia during the early 1980s [23]. Between 2013 and 2014, PEDV epidemics spread from the United States to neighboring North American countries and quickly extended globally [24,25,26,27]. Currently, PEDV is recognized as a significant global health concern. PDCoV was initially discovered in 2012 in pig fecal samples during a coronavirus surveillance study in Hong Kong [28]. PDCoV infections have been reported in the U.S. and China in 2014, with substantial economic and safety concerns for the pig industry [29]. Studies indicate that PDCoV can infect animal-derived cell lines and achieve cross-species transmission; potential infection hosts include chickens, cattle, and even human beings [30,31,32]. TGEV was first discovered in the U.S. in 1946 and subsequently reported in countries across Europe, Asia, Africa, and the Americas [33]. In recent years, TGEV has been observed to be recombining and evolving continuously, and some of these recombinant strains can spread across species, making the detection and prevention of TGEV more complex [34,35].

These viruses share similar pathogenic mechanisms, damaging intestinal epithelial cells and villi and leading to nutrient malabsorption and severe diarrhea, which can be fatal due to dehydration and shock, especially in piglets. Given the high detection rates of porcine coronaviruses and their frequent co-infection with other pathogens, coupled with their propensity for mutation and recombination, accurate and sensitive detection methods are crucial to differentiate them from other pathogens and manage outbreaks effectively. In order to effectively monitor these porcine CoVs, studies have reported a variety of nucleic acid detection technologies, such as multiplex PCR, isothermal methods (LAMP, NASBA, RPA, and PSR), CRISPR-Cas, and microfluidics platforms, in recent years [36]. Among these, the most widely used method is undoubtedly the PCR-based method. The relatively economical RT-PCR method has detection limits for PDCoV, PEDV, TGEV, and SADS-CoV of 5.66 × 10^5^ copies/μL, 6.48 × 10^5^ copies/μL, 8.54 × 10^5^ copies/μL, and 7.79 × 10^6^ copies/μL, respectively [37]. The more sensitive quadruple real-time quantitative PCR method directly lowers the limit of detection to 1 × 10^2^ copies/µL [17,38]. To enhance the reliability of the qPCR assay, a five-plex qPCR incorporating an external positive control, namely XIPC RNA, at the nucleic acid extraction stage, was established. This assay was subsequently applied to analyze 219 clinical samples collected from the U.S., thereby providing robust support for the epidemiological investigation of PEDV, PDCoV, TGEV, and SADS-CoV [39].

Droplet digital PCR (dPCR) provides several technological benefits, including excellent anti-interference abilities, high accuracy, and sensitivity. It uses droplet generation technology, similarly to microfluidics, to divide the PCR reaction system. It uses two mutually immiscible liquids to form tiny droplets through the interaction between the two surfaces. The droplet production apparatus can produce millions of nanoliter-sized water-in-oil droplets as sample dispersion carriers. Under ideal circumstances, each droplet can contain a single copy of the nucleic acid template and a single microsystem reaction solution for amplification. Finally, the target nucleic acid is precisely measured by measuring the droplet’s fluorescence signal; particular primers and probes are included in the system, allowing for viral differential diagnosis.

The multiplex porcine enteric coronavirus dPCR assay, established for the first time in this study, has strong specificity, good stability, reproducibility, and high sensitivity. It can quantify the copy concentrations of four target viruses in actual samples, and we use the multiplex dPCR detection method to simplify the number of test operations, enable the simultaneous detection of multiple targets, reduce operational errors and consumption, and reduce the detection costs to a certain extent. At the same time, this method can meet the requirements of qualitative and quantitative virus identification in samples, as well as providing a new and reliable assay for the early identification and monitoring of the four viruses in the early stages of infection, differential diagnosis with other common viruses, the study of their transmission patterns, epidemiological risk assessment, and other systematic applications.

## 2. Result

### 2.1. PCR Reaction Optimization

**Figure 1:**
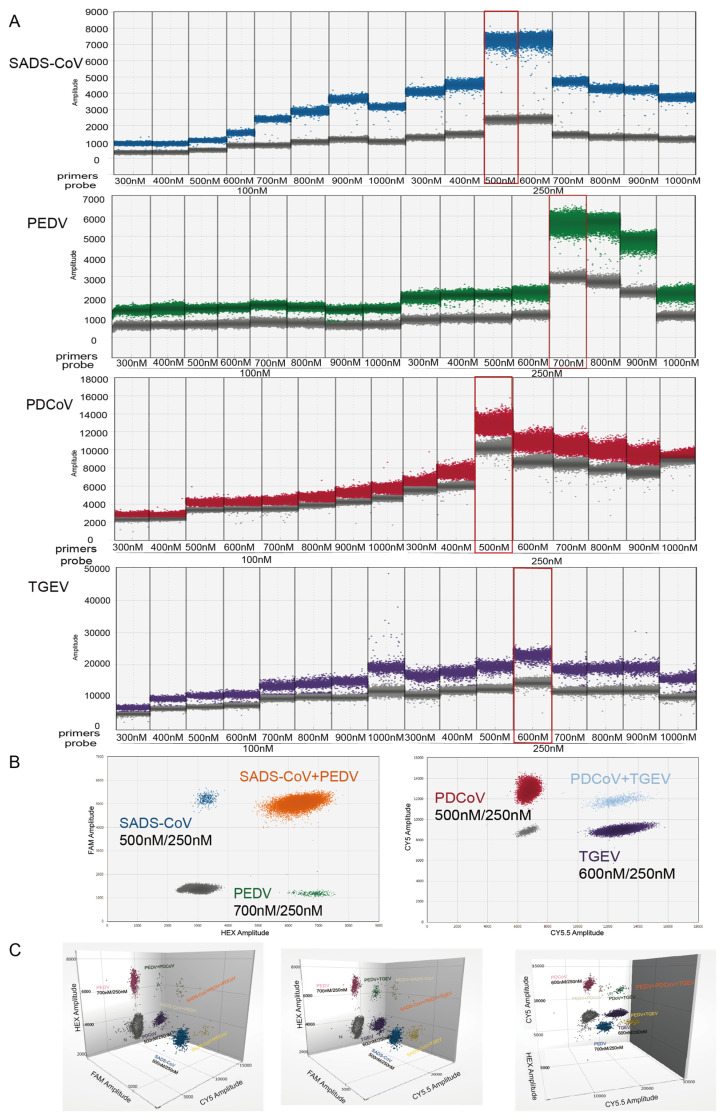
Optimization of concentrations in dPCR system. The primer and probe concentrations were optimized from 300 to 1000 nM and 250 to 900 nM, respectively. Four target standard plasmids (10^3^ copies/reaction) were used in this optimization. Positive results are indicated in color. (**A**) Single fluorescence channel result (1D diagram) of multiple dPCR for various primer/probe combinations; the optimal primer probe concentration is indicated by a red box. (**B**) Double fluorescence channel result (2D diagram); the optimized concentrations of the primer/probe are labeled under the target virus name. (**C**) Triple fluorescence channel result (3D diagram). All grey color dot represent negative.

**Figure 2:**
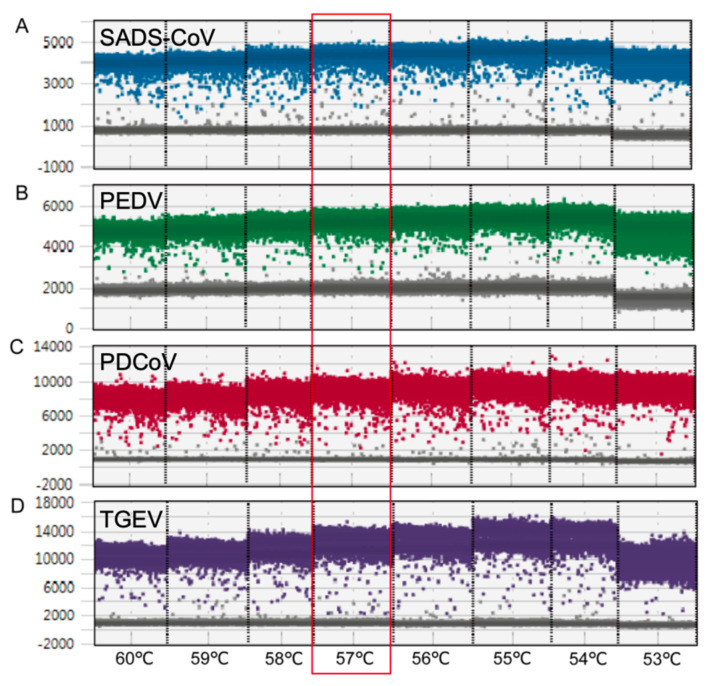
Optimization of the annealing temperature of the dPCR system. Using 10^3^ copies/reaction of (**A**) SADS-CoV, (**B**) PEDV, (**C**) PDCoV, and (**D**) TGEV positive plasmids, the annealing temperature for the dPCR primers was optimized from 60 to 53 °C. By comparing the negative and positive differences and the number of drops, the optimal annealing temperature was determined to be 57 °C (red box).

Multiplex dPCR detection was optimized by assessing the concentration ranges of primers (300 to 1000 nM) and probes (100 to 250 nM) for each virus’ detection. The red boxes marked in Figure 1 indicate the optimal concentrations of primers and probes for SADS-CoV (500 nM/250 nM), PEDV (700 nM/250 nM), PDCoV (500 nM/250 nM), and TGEV (600 nM/250 nM). When the optimal primer annealing temperature was 57 °C (Figure 2), the fluorescence value of the positive droplets was relatively high. The negative and positive microdroplets were concentrated, the number of scattered microdroplets in the middle was small, and the clusters of positive microdroplets of each virus showed obvious and clear zoning. The optimal reaction system for dPCR was determined as shown in Figure 1, and the optimal reaction conditions were as follows: 50 °C for 20 min; 95 °C for 10 min; 94 °C for 30 s, 57 °C for 1 min, 40 cycles; 98 °C for 10 min; 4 °C hold. All reactions were performed at a lift rate of 2 °C/s. Figure 1B shows a schematic diagram of the 2D reaction when all four target viruses were positive under the optimal concentration of the primer probe, and Figure 1C shows the 3D schematic diagram when all four target viruses were positive at the optimal primer and probe concentrations.

### 2.2. Anti-Interference

**Figure 3:**
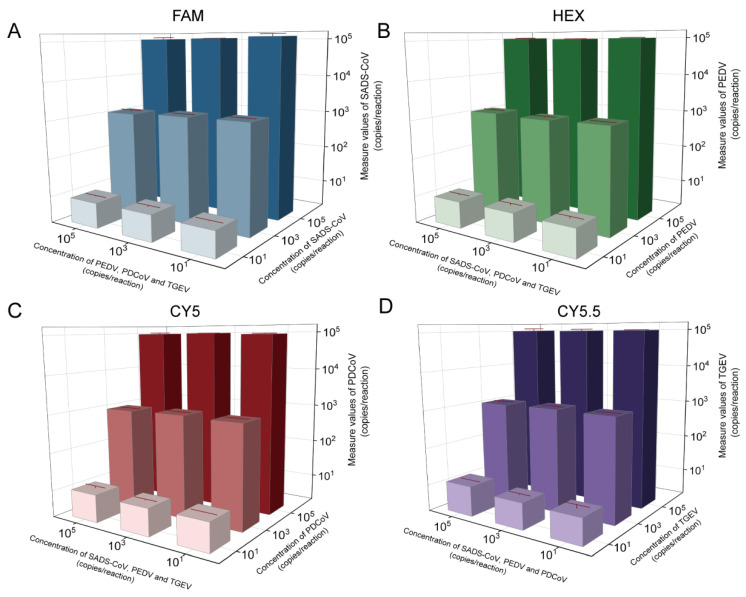
Interference resistance of multiple dPCR. (**A**) SADS-CoV-, (**B**) PEDV-, (**C**) PDCoV-, and (**D**) TGEV-positive plasmid mixtures (10^1^ to 10^5^ copies/reaction) were detected by multiple dPCR, respectively. X- and Y-axes: concentrations of positive plasmids; Z-axis: measured values of target.

Four virus-positive plasmids with a high concentration (1.0 × 10^5^ copies/reaction), medium concentration (1.0 × 10^3^ copies/reaction), and low concentration (1.0 × 10^1^ copies/reaction) were combined to evaluate whether the concentration of each target of the method would affect the quantification of the target, and the assays of each group were repeated individually three times. The results showed that, regardless of the concentrations of other viruses in the system, they did not affect the detection of the target virus, which verified that the developed method was highly resistant to interference (Figure 3 and Figure S1–S4).

### 2.3. Specificity of the Multiplex dPCR

**Figure 4:**
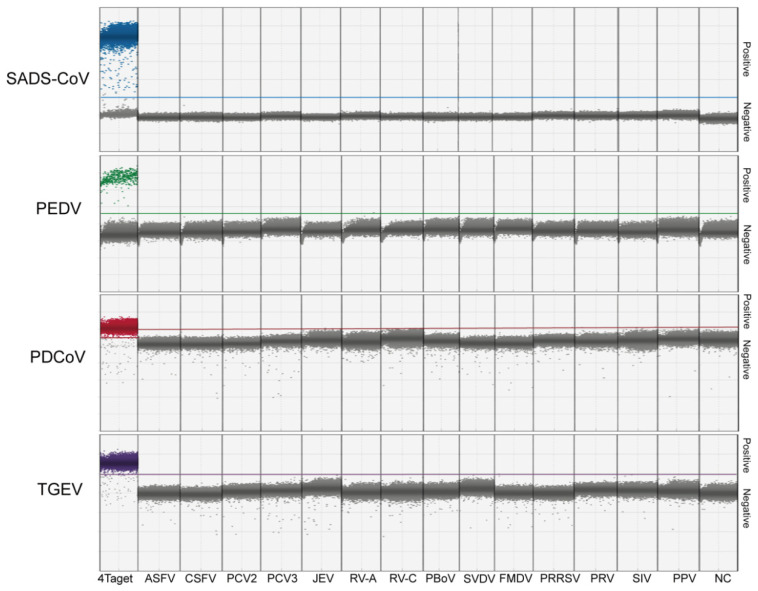
The specificity of dPCR. The cDNA mixture of the 4 viruses (SADS-CoV, PEDV, PDCoV, and TGEV), the DNA extraction of 6 DNA viruses (ASFV, PCV2, PCV3, PBoV, PRV, PPV), the cDNA of 9 RNA viruses (CSFV, JEV, RV-A, RV-C, JEV, SVDV, FMDV, PRRSV, SIV), and a negative control (ddH_2_O) were assessed by this multiple dPCR, respectively. The positive reads are colored in blue (SADS-CoV), green (PEDV), red (PDCoV), and purple (TGEV). The negative reads are colored in grey.

SADS-CoV, PEDV, PDCoV, and TGEV cDNA, with other common swine viruses’ DNA/cDNA, including ASFV, CSFV, PCV2, PCV3, JEV, RV-A, RV-C, PBoV, SVDV, FMDV, PRRSV, PRV, SIV, and PPV, were used as templates, and ddH_2_O was used as a negative control, to conduct a multiplex dPCR in order to evaluate the specificity of the assay. The specificity results showed that only the positive samples of SADS-CoV, PEDV, PDCoV, and TGEV showed specific amplification, while the other test items showed no positive fluorescence signals and were negative. This indicated the good specificity of the assay, with no cross-reaction with common viruses (Figure 4).

### 2.4. Repeatability and the Standard Curve

**Figure 5:**
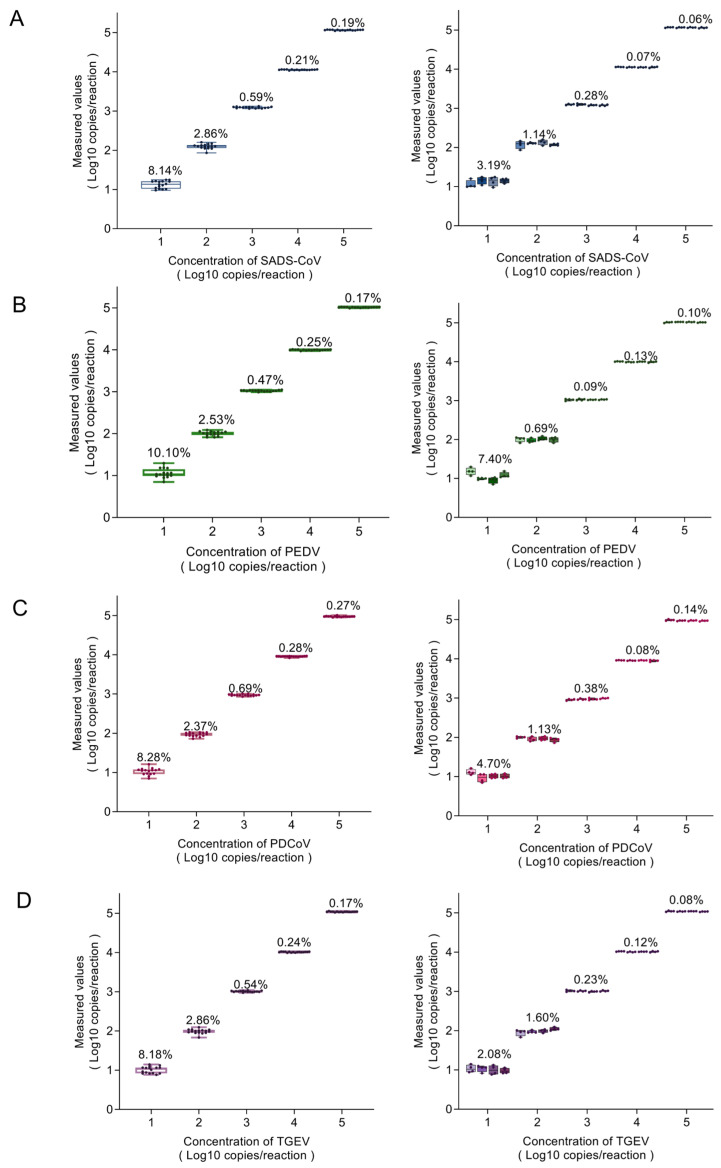
Intra- and inter-batch repeatability of the multiplex digital PCR. The standard plasmids of (**A**) SADS-CoV, (**B**) PEDV, (**C**) PDCoV, and (**D**) TGEV, with copy numbers varying from 10^1^ to 10^5^ copies/reaction, were detected by the established dPCR. Left: intra-batch repeatability with five concentrations (n = 16). Right: inter-batch repeatability with five concentrations at four different time points (n = 4). X-axis: plasmid concentrations, Y-axis: measured values. Error bars represent the SD, and numerical values represent the CVs.

The positive plasmids of SADS-CoV, PEDV, PDCoV, and TGEV with five concentrations (10^5^ to 10^1^ copies/reaction) were used as templates for the dPCR reaction; 16 replicates were set for each dilution, and the corresponding CVs of each dilution were calculated. The results showed that the intra-batch repeat CV%s of SADS-CoV were 0.19–8.14%; the intra-batch repeat CV%s of PEDV were 0.17–10.10%; the intra-batch repeat CV%s of PDCoV were 0.27–8.28%; and the intra-batch repeat CV%s of TGEV were 0.17–8.18%. All were less than 11%. The above plasmids were tested at four different times, four replicates were set for each dilution, and the CVs of each dilution were calculated for the four batches of independent experiments. The results showed that the CV%s of the inter-batch repeats for SADS-CoV were 0.06–3.19%; the CV%s of the inter-batch repeats for PEDV were 0.09–7.40%; the CV%s of the inter-batch repeats for PDCoV were 0.08–4.70%; and the inter-batch repeat CV%s for TGEV were 0.08–2.08%. All were less than 8%. Within the assay range, the inter- and intra-batch repeat CV%s for all four viruses were less than 11%, and the method showed high repeatability (Figure 5).

**Figure 6:**
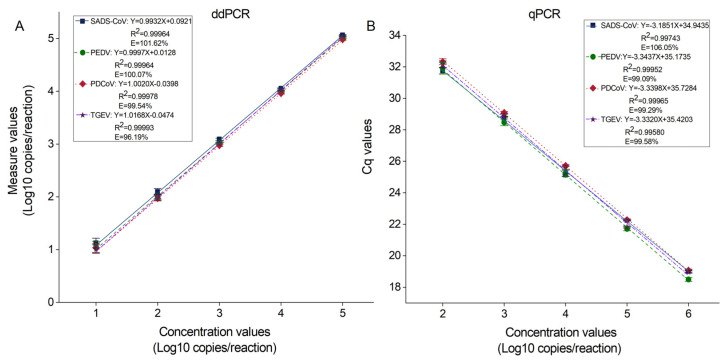
Standard curves of dPCR and qPCR. The positive plasmids of 4 virus concentrations from 10^5^ to 10^1^ copies/reaction were used to establish a standard amplification curve. (**A**) The standard curves of ddPCR. X-axis: theoretical plasmid concentration; Y-axis: actual measured concentration of dPCR. (**B**) The standard curves of qPCR. X-axis: theoretical plasmid concentration; Y-axis: Cq values measured by qPCR. Data represented as means ± SD of 16 replicates. R^2^: coefficient of determination. E: amplification efficiency.

The standard curve was plotted according to the results of 16 repetitions (Figure 6) and compared with that of a previously reported qPCR method [17], which showed that the amplification efficiency (E) of the four target viruses with the developed method was in the range of 90–110%, which was good, and the R^2^ was greater than 0.999. This indicates that the actual detection value fits well with the theoretical curve and shows the high stability of the detection result. The E and R^2^ are comparable with those of the qPCR, but, at a 10^1^ copies/reaction concentration gradient, some of the 16 replicates could not be detected by qPCR.

### 2.5. Sensitivity of the Multiplex dPCR

**Figure 7:**
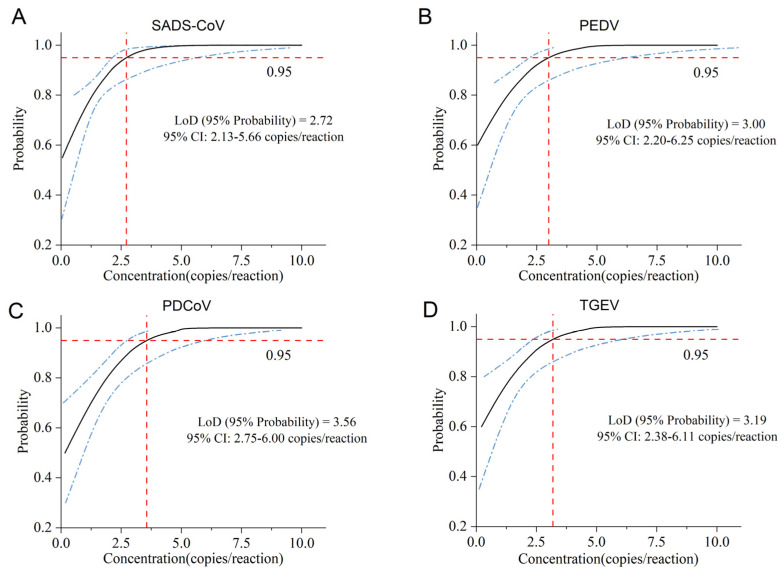
Probit analysis sigmoid curve reporting the LoD of dPCR. The positive plasmids of (**A**) SADS-CoV, (**B**) PEDV, (**C**) PDCoV, and (**D**) TGEV, at concentrations from 1 × 10^0^ to 1 × 10^3^ copies/reaction, were used, and 40 replicates were performed for each concentration. The limit of detection (LoD) of the assay was calculated within the 95% confidence interval (CI) according to the probit regression model with a reproducible probability of 95% to assess the sensitivity of the assay. X-axis: expected concentration (copies/reaction). Y-axis: fraction of positive results in all parallel reactions performed. The black line is a probit curve. The blue lines are 95% confidence intervals (95% CIs). The red cross lines represent 95% probability of LoD.

The sensitivity test was carried out using a standard plasmid with a theoretical concentration of 1 × 10^3^–10^0^ copies/reaction. The lowest concentration that could be detected with relative standard deviation (RSD) ≤ 25% in 40 replicates was the limit of quantification (LoQ) of the method. According to the detection results and RSD (Table 1), the LoQ of the multiplex dPCR was 7.5 copies/reaction; the LoQ of the qPCR was 10^2^ copies/reaction, since the qPCR detected no Cq value at 1 × 10^1^ copies/reaction. To further accurately determine the LoQ of dPCR, according to the probit regression model with a repeatability probability of 95% (Figure 7), the LoDs for SADS-CoV, PEDV, PDCoV, and TGEV were calculated as 2.72 copies/reaction (95%CI: 2.13–5.66 copies/reaction), 3.00 copies/reaction (95%CI: 2.20–6.25 copies/reaction), 3.56 copies/reaction (95%CI: 2.75–6.00 copies/reaction), and 3.19 copies/reaction (95%CI: 2.38–6.11 copies/reaction), respectively. Moreover, the sensitivity of dPCR was one order of magnitude higher than that of qPCR.

### 2.6. Sample Detection of dPCR and qPCR

**Figure 8:**
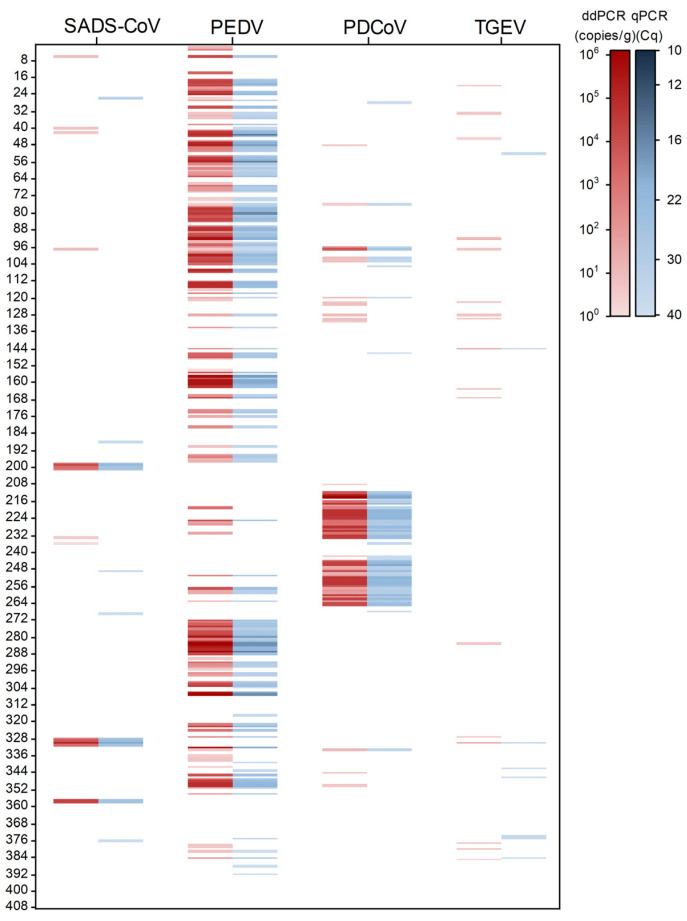
A total of 408 samples were analyzed by dPCR (red) and qPCR (blue). The mean of the concentration (copies/g) or Cq value for each sample is scaled with red and blue colors, respectively.

**Figure 9:**
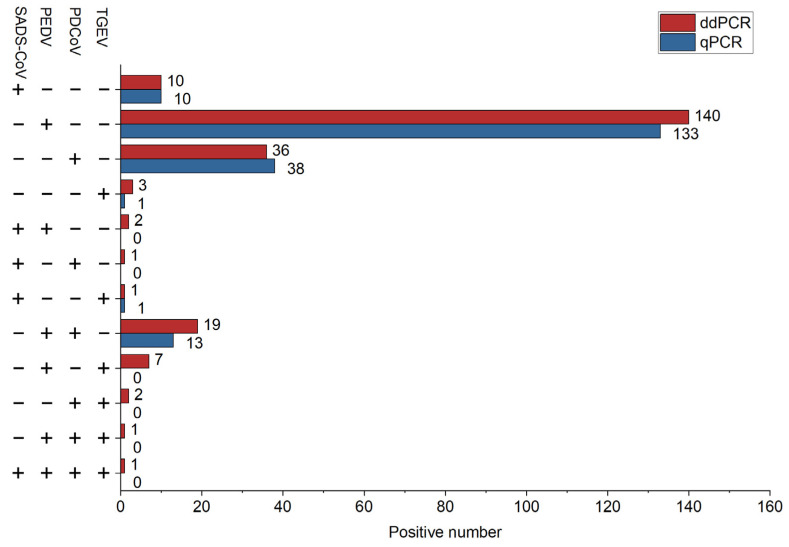
Comparison of positive detection for dPCR and qPCR. Number of positive detections for each pathogen in 408 samples using dPCR (red) and qPCR (blue).

In total, 408 samples were analyzed by the established dPCR to quantify the viral concentrations in the samples, and the results were compared with those of the qPCR assay, as shown in Figure 8. The colored concentration represents the viral concentration in the sample, and the results of the two methods are the same; 223 positive samples and 185 negative samples were detected by the dPCR assay. In particular, 34 of the positive samples were mixed infections of two or more viruses, among which the number of mixed infections of PEDV and PDCoV was the highest, and there were four viruses infecting at the same time (Figure 9). The proportion of mixed infections in positive samples was 15.00% in tissue, 17.28% in feces, and 12.50% in serum samples, while no mixed infections were detected in feed samples (Table 2).

A comparison with known results (Table 3) revealed that the negative detection results obtained by the established dPCR and qPCR assays exhibited good consistency with the known outcomes. For each target virus, the compliance rate (i.e., diagnostic specificity, DSp) ranged from 97% to 100%, and the samples involved had been previously validated using the commercial qPCR kit. However, among the four sample categories, the number of false positive results detected by dPCR was only one to two, corresponding to diagnostic sensitivity (DSe) of 97% to 100%. In contrast, qPCR showed a significantly higher number of false positives (at least three-fold) for each target virus, with its DSe ranging from 13% to 85%. Notably, in feed and fecal samples, dPCR successfully detected target pathogens at the 10^0^ copies/reaction magnitude, whereas qPCR yielded a negative result (no Cq values). These results demonstrate that, when analyzing samples with complex matrices, dPCR exhibits higher sensitivity and accuracy than qPCR. Additionally, dPCR can stably quantify the number of viral copies within the detection range of the assay.

## 3. Discussion

SADS-CoV, PEDV, PDCoV, and TGEV are all highly infectious and lethal porcine enteric CoVs that significantly impact the swine industry. Infected piglets typically display gastrointestinal, respiratory, and neurological symptoms, leading to a range of diseases associated with porcine viral diarrhea [40]. Co-infections involving two or more of these viruses have been frequently reported across various pig farms [26,41,42,43]. This may be due to the high rates of infection with these viruses. For instance, a study estimated that the prevalence of PDCoV infection in China was 12.2% [44]. In addition, these viruses can interact with host proteins to regulate pathways and effectively inhibit the host immune response, thereby promoting viral replication and co-infection. SADS-CoV exerts effects on host cells, including the induction of apoptosis through exogenous death receptors and endogenous mitochondrial pathways, as well as the induction of autophagy via the IRE1-JNK-Beclin1 and AKT/mTOR signaling pathways [45]. Another study found that PDCoV and PEDV co-infection regulated pro-inflammatory cytokines through the TRAF6-mediated canonical NF-κB and IRF7 signaling pathways, leading to enhanced viral evasion of the mucosal innate immune response [46]. Since it is difficult for clinical diagnoses to distinguish between them, there is a need for accurate and sensitive diagnostic tools.

Although dPCR is regarded as an iteration of qPCR, mainstream research at present still focuses on comparisons between these two methods. Our study demonstrated that the established dPCR assay is capable of differentiating and identifying the SADS-CoV, PEDV, PDCoV, and TGEV in a single reaction, showcasing high specificity and resistance to interference. The dPCR method also showed high amplification efficiency and reproducibility, with a CV of intra- and inter-batch repeats of less than 11%, which is comparable to that of the qPCR method (Figure 5 and Figure 6). This is primarily attributed to dPCR dividing the reaction system into numerous small independent reaction units, which reduces the impact of reaction inhibitors and primers, thereby enhancing the consistency and stability of amplification.

The LoQ and LoD of the established dPCR assay are superior to those of qPCR, with dPCR achieving an LoQ of 7.5 copies/reaction (Table 1) and an LoD of less than 5 copies/reaction (Figure 7), an order of magnitude better than qPCR. This aligns with previous studies that have reported that dPCR has high sensitivity and precision in detecting low-abundance nucleic acids [47]. However, our study also identified the upper limit of detection as a limitation, requiring sample dilution for concentrations above 10^6^ copies/reaction. This is consistent with the known limitations of dPCR platforms, suggesting the need for further refinement of the assay to accommodate a broader dynamic range.

To verify the applicability of the established method in real samples, sample validation tests were performed on 408 known samples, and the results were consistent with those of pre-determined samples, with a 97–100% compliance rate (Table 3). The Dsp of the assay was 99–100%, which was slightly better than that of qPCR (97–99%). Interestingly, the Dse of qPCR for the detection of SADS-CoV and TGEV was only 67% and 13%, respectively, compared with 100% for both when using dPCR (Table 2). These results indicate that dPCR is more advantageous in detecting these two viruses.

While the dPCR assay demonstrated high specificity and sensitivity, the potential for false positives due to cross-contamination during sample preservation is a concern, especially given its high sensitivity. As shown in Table 3, the false positive rate of dPCR in the complex matrix was 1.47% (6/408), which was less than the value of 6.62% (27/408) for qPCR, demonstrating that our method has stable reliability and can achieve good sensitivity and specificity in the analysis of real samples.

This study, through a comparison with qPCR, demonstrates that digital PCR, as a highly sensitive and highly specific detection technology, shows promising potential for practical applications. However, it also presents certain limitations. First, the droplet generation step in digital PCR has a relatively low throughput, typically allowing the processing of only 8 to 16 samples per experiment, which reduces its convenience for large-scale screening. Second, compared with qPCR, digital PCR’s equipment and reagents are relatively expensive [48], requiring a specialized droplet generation oil to ensure the formation of independent reaction units, thereby increasing the cost of detection. This also makes the operation relatively complex, particularly since the droplet generation process is susceptible to environmental and personnel influences, placing higher technical demands on operators. In addition, when detecting high-concentration amplification products, the limited number of generated droplets restricts the upper limit of the dynamic range, thus limiting its applicability in high-concentration samples [49,50].

Despite these limitations, digital PCR still offers irreplaceable advantages in specific applications, such as low-concentration detection and the testing of samples with complex matrices. In the short term, digital PCR and qPCR will likely play complementary roles in different directions and application fields within pathogen detection. With continued technological development, cost-related issues are expected to be alleviated, as seen with qPCR, while the upper detection limit may be addressed by improving and optimizing the droplet generation technology to increase the droplet quantity and quality. If these challenges are resolved, digital PCR has the potential to truly replace qPCR as the mainstream molecular biology detection method.

## 4. Materials and Methods

### 4.1. Virus

Adenovirus containing the entire *p72* gene of the African swine fever virus (ASFV) was purchased from Sangon (Shanghai, China). Foot-and-mouth disease virus (FMDV) inactivated vaccine was purchased from Jinyu Biotechnology (Inner Mongolia, China). Classical swine fever virus inactivated vaccine (CSFV, C strain) and swine influenza virus inactivated vaccine (SIV, TJ strain) were purchased from Keqian Biotechnology (Wuhan, China). Swine acute diarrhea syndrome coronavirus (SADS-CoV) was donated by Tianjin Customs; porcine reproductive and respiratory syndrome virus (PRRSV), porcine deltacoronavirus (PDCoV), porcine epidemic diarrhea virus (PEDV) (CV777 strain), pseudorabies virus (PRV, Bartha strain), and porcine circovirus type 2 (PCV2, JH SRJ strain) were donated by Zhejiang University (Hangzhou, China); porcine parvovirus (PPV)-positive samples were donated by Zhejiang A&F University (Hangzhou, China). Positive Japanese encephalitis virus (JEV), porcine bocavirus (PBoV), transmissible gastroenteritis virus (TGEV), porcine rotavirus A/C (RV-A/C), and swine vesicular disease virus (SVDV) samples were preserved by our laboratory.

### 4.2. Primers and Probes

**Figure 10:**
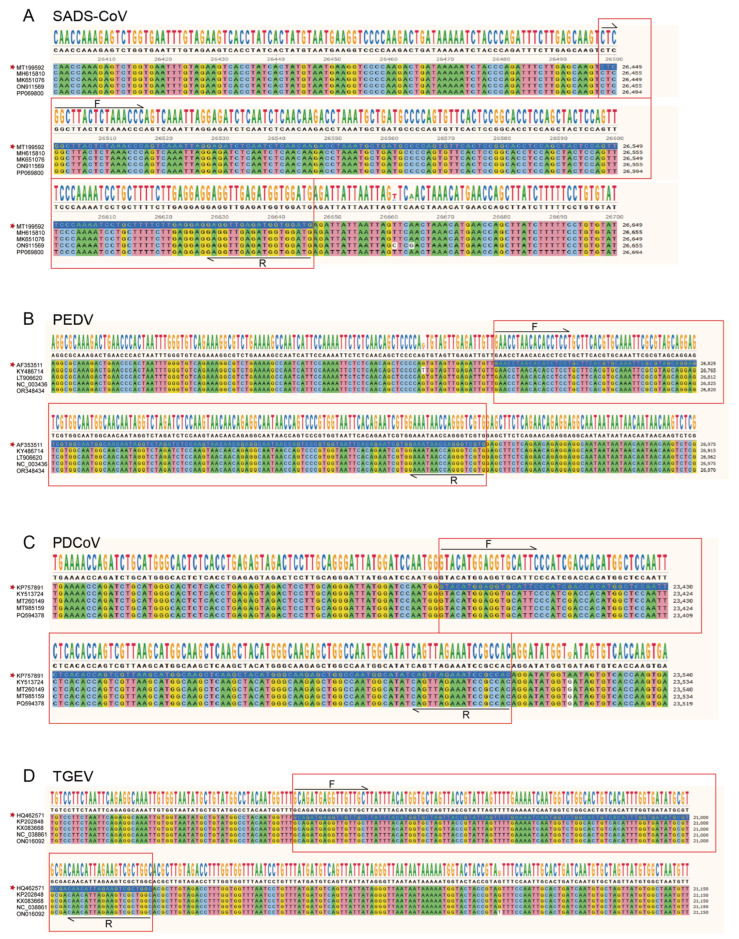
The binding regions of the four coronavirus primers were highly conserved. Five sequences of the target genes of (**A**) SADS-CoV, (**B**) PEDV, (**C**) PDCoV, and (**D**) TGEV were selected for comparison, respectively. The red box showed that the conservation of all primer target sequences was 100%. GenBank accession numbers for alignment sequences were displayed in front of the sequences.

Four pairs of specialized primers and probes were designed using Primer3Plus, version 3.3.0, based on the conserved sequences of the SADS-CoV *N* gene (MT199592), PEDV *N* gene (AF353511.1), PDCoV *M* gene (KP757891), and TGEV *S* gene (HQ462571). As shown in Figure 10, sequence alignment ensured that all of the primer and probe target region sequences were highly conserved. The primers and probes used in this technical assay were synthesized by Sangon Biotechnology (Shanghai, China) and subsequently used to develop the multiplex dPCR. The specific primers and probes used are listed in Table 4.

### 4.3. Standard Plasmid Construction and Concentration Determination

According to the conserved gene sequences selected from each target virus in GenBank as templates, the fragments were amplified with the primers shown in Table 4. Positive plasmids were constructed by Sangon (Shanghai, China), and their concentrations were determined after dilution with ddH_2_O. According to the formula copy number concentration (copy/reaction) = [6.02 × 10^23^ × (plasmid concentration ng/μL × 10^−9^)] × reaction volume (20 μL)/[DNA length (bp) × 660], we calculated the copy number concentration of the recombinant plasmid, determined its initial concentration at 1 × 10^8^ copies/reaction, diluted it with nuclease-free water in a concentration gradient according to certain proportions, and stored it at −20 °C, after which it was subjected to different experiments.

### 4.4. Nucleic Acid Extraction and Reverse Transcription

Nucleic acids of all samples and viruses were extracted using the Viral DNA and RNA Extraction Kit 4.0 (Tianlong, Anshun, China), as described in the kit instructions. The extracted nucleic acids were reverse-transcribed into cDNA with Prime Script IV 1st Strand cDNA Synthesis Mix (TaKaRa, Kusatsu, Japan) and stored at −20 °C.

### 4.5. Optimization of the Multiplex dPCR Assay

The positive plasmid of each target virus at a 10^3^ copies/reaction concentration was used as the reaction template, and the concentrations of the primers and probes were optimized using the QX600^TM^ Droplet Digital PCR System (Bio-Rad, Hercules, CA, USA). The results for different combinations of different primer concentrations (300 to 1000 nM) and different probe concentrations (100 to 250 nM) of each target were analyzed to determine the optimal matching of primers and probes. After determining the optimal matching of the primers and probes, the temperature range of 53–60 °C was set to optimize the annealing temperature. The final optimization of the reaction conditions of the system was achieved by analyzing the 1D, 2D, and 3D plots of each combined reaction system and comparing the number of droplets generated, the dispersion state of the droplets, the distribution of negative and positive droplets, and the intensity of the fluorescence signals of the droplets with other indicators.

### 4.6. Anti-Interference Analysis

We combined the diluted positive plasmids of the four viruses at a ratio of 1:1:1:1 to prepare 36 different mixed samples to be detected. These samples included high concentrations (1 × 10^5^ copies/reaction), medium concentrations (1 × 10^3^ copies/reaction), and low concentrations (1 × 10^1^ copies/reaction). Each group was assayed in triplicate to evaluate the interference resistance of the method.

### 4.7. Specificity, Repeatability, and Sensitivity of the Multiplex dPCR

The cDNA of SADS-CoV, PEDV, PDCoV, and TGEV was prepared via a cDNA synthesis kit, and the concentrations of the four viral cDNA samples were 1.89 × 10^4^, 1.62 × 10^3^, 1.52 × 10^5^, and 1.51 × 10^5^ copies/reaction, respectively. Then, the cDNA of SADS-CoV, PEDV, PDCoV, and TGEV, together with other common porcine virus’ DNA or cDNA, such as ASFV, CSFV, PCV2, PCV3, JEV, RV-A, RV-C, JEV, PBoV, SVDV, FMDV, PRRSV, PRV, SIV, and PPV, was used as dPCR templates, while ddH_2_O was used as a negative control, to evaluate the specificity of the assay.

The positive plasmids of SADS-CoV, PEDV, PDCoV, and TGEV were diluted 10-fold (10^5^ to 10^1^ copies/reaction) for the dPCR assay, and 16 replicates were set up for each dilution. The corresponding coefficients of variation (CVs) of each dilution were calculated to evaluate the intra-batch repeatability of the assay. The above plasmids were tested at four different times, and four replicates were set for each dilution. The CVs of each dilution for four batches of independent experiments were calculated to evaluate the inter-batch repeatability of the assay.

The 10-fold multiplicity dilution of 10^1^–10^6^ copies/reaction of the mixed positive plasmid was used as the template for the dPCR assay, and 16 replicates were set for each concentration. According to the actual measured concentration, the standard curve was plotted using Origin (OriginLab Corporation, Northampton, MA, USA). Meanwhile, the assay was compared with the multiplexed qPCR method that was used in a previous study [17], and the correlation coefficients (R^2^) of the two methods were calculated to assess the amplification efficiency (E) of the two systems.

To further determine the sensitivity of our dPCR method, sensitivity tests were performed with the theoretical concentration of 10^0^–10^3^ copies/reaction of the positive plasmid, and 40 replicates were performed for each concentration. The lowest concentration that could be detected in all 40 replicates with relative standard deviation (RSD) ≤ 25% was designated as the limit of quantitation (LoQ) of the assay. To obtain a more accurate limit of detection (LoD) for the dPCR system, the LoD of the assay was calculated within the 95% confidence interval (CI) according to the probit regression model with a reproducible probability of 95% to assess the sensitivity of the assay.

### 4.8. Sample Detection

To validate the present method, the established multiplex dPCR assay for porcine enteric coronavirus was employed, with 408 known negative and positive samples serving as the validation materials. These samples were collected in Zhejiang Province, China, from 2023 to 2024, and stored in our laboratory. Moreover, they were previously verified using commercial pathogen detection qPCR kits (Zhonglianrui, Beijing, China). Subsequently, the virus concentrations in the above samples were quantified via the established multiplex dPCR assay. For the purpose of evaluating the accuracy and stability of this method in practical sample detection, the quantification results were compared with those obtained from the multiplex qPCR assay. The samples used in this comparative analysis covered four types of samples, namely porcine digestive tract tissue (n = 210), feces (n = 130), serum (n = 41), and feed (n = 36).

## 5. Conclusions

The multiplex dPCR assay developed in this study for porcine enteric coronaviruses exhibits high specificity, stability, reproducibility, and sensitivity, making it an ideal tool for the early detection and tracing of porcine viral diarrhea syndrome pathogens. The multiplex capability of this dPCR assay reduces the cost and errors associated with multiple testing, and its direct quantification of the virus concentration enhances the comparability of the results across laboratories. This study not only provides a new and reliable method for the differential diagnosis of these viruses but also contributes to the understanding of their transmission patterns and the systematic application of epidemiological risk assessment.

## Figures and Tables

**Table 1:** Alignment of dPCR with qPCR: sensitivity.

Positive Plasmid *		dPCR (n = 40)			qPCR (n = 40)		
		Mean ± SD *	Positive	RSD ^†^	Mean Ct ± SD *	Positive	RSD ^†^
SADS-CoV	10^3^	1235.38 ± 51.82	40	0.56%	28.76 ± 0.08	40	0.27%
	10^2^	126.25 ± 16.64	40	2.23%	31.74 ± 0.20	40	0.62%
	10^1^	13.02 ± 4.74	40	8.14%	/	39	/
	7.5	9.75 ± 3.69	40	18.55%	/	35	/
	5	7.70 ± 3.5	40	31.40%	/	19	/
	2.5	4.59 ± 2.35	40	40.67%	/	4	/
	1	/	32	/	/	0	/
PEDV	10^3^	1059.63 ± 34.58	40	0.47%	28.47 ± 0.21	40	0.75%
	10^2^	101.48 ± 1.57	40	2.53%	31.91 ± 0.37	40	1.16%
	10^1^	12.65 ± 4.81	40	10.10%	/	38	/
	7.5	8.58 ± 3.43	40	21.48%	/	31	/
	5	5.66 ± 2.16	40	27.20%	/	16	/
	2.5	/	37	/	/	10	/
	1	/	29	/	/	0	/
PDCoV	10^3^	939.25 ± 44.18	40	0.69%	29.10 ± 0.07	40	0.22%
	10^2^	93.89 ± 9.81	40	2.37%	32.33 ± 0.18	40	0.57%
	10^1^	9.75 ± 3.56	40	8.28%	/	34	/
	7.5	6.881 ± 2.22	40	19.16%	/	32	/
	5	4.66 ± 2.37	40	46.33%	/	20	/
	2.5	/	34	/	/	3	/
	1	/	27	/	/	0	/
TGEV	10^3^	1020.88 ± 38.27	40	0.54%	28.90 ± 0.10	40	0.36%
	10^2^	98.40 ± 12.34	40	2.86%	32.17 ± 0.21	40	0.65%
	10^1^	11.39 ± 2.77	40	11.35%	/	39	/
	7.5	7.41 ± 2.57	40	18.19%	/	34	/
	5	4.96 ± 1.54	40	20.80%	/	13	/
	2.5	/	36	/	/	6	/
	1	/	29	/	/	0	/

*: The unit of the concentration is copies/reaction. ^†^: Relative standard deviation.

**Table 2:** The results of 408 samples under dPCR and qPCR.

Sample Type	Target Virus				dPCR		qPCR	
	SADS-CoV	PEDV	PDCoV	TGEV	Positive	Multiple-Pathogen Detection Rate	Positive	Multiple-Pathogen Detection Rate
Intestinal tissue (n = 201)	+	−	−	−	5	15.00% (18/120)	4	6.73% (7/104)
	−	+	−	−	94		92	
	−	−	+	−	2		/	
	−	−	−	+	1		1	
	+	+	−	−	2		/	
	−	+	+	−	7		7	
	−	+	−	+	5		/	
	−	−	+	+	2		/	
	−	+	+	+	1		/	
	+	+	+	+	1		/	
Feces (n = 130)	+	−	−	−	3	17.28% (14/81)	4	8.86% (7/79)
	−	+	−	−	31		31	
	−	−	+	−	33		37	
	+	−	+	−	1		/	
	+	−	−	+	1		1	
	−	+	+	−	10		6	
	−	+	−	+	2		/	
Serum (n = 41)	+	−	−	−	2	12.50% (2/16)	2	0% (0/11)
	−	+	−	−	11		8	
	−	−	+	−	1		1	
	−	+	+	−	2		/	
Feed (n = 36)	−	+	−	−	4	0% (0/6)	2	0% (0/2)
	−	−	−	+	2		/	
Total (n = 408)					223	15.25% (34/223)	196	7.14% (14/196)

**Table 3:** Alignment of DSp and DSe between dPCR and qPCR.

	Target Virus:	Sample Number (n = 408)				Number of Positive Detections							
						dPCR				qPCR			
	SADS-CoV	+				+				+			
	PEDV		+				+				+		
	PDCoV			+				+				+	
	TGEV				+				+				+
Tissue	Positive	8	110	14	10	8	110	13	10	4	99	7	1
Feces		5	44	45	3	5	43	44	3	4	37	43	1
Serum		2	13	3	0	2	13	3	0	2	8	1	0
Feed		0	5	0	2	0	4	0	2	0	2	0	0
Total		15	172	62	15	15	170	60	15	10	146	51	2
Tissue	Negative	193	91	187	191	0	1	0	1	2	1	3	1
Feces		125	86	85	127	1	0	1	0	2	2	4	0
Serum		39	28	38	41	0	1	0	0	0	2	0	2
Feed		36	31	36	34	0	0	0	1	1	3	1	3
Total		393	236	346	393	1	2	1	2	5	8	8	6
DSp * (%)						100	99	100	99	99	97	98	98
DSe ^†^ (%)						100	99	97	100	67	85	82	13

*: Diagnostic specificity, DSp = true negative/(true positive + true negative) × 100%. ^†^: Diagnostic sensitivity, DSe = true positive/(true positive + true negative) × 100%.

**Table 4:** Primers and probes used in this study.

Primer/Probe		Sequence (5′ to 3′)	Position	Product (bp)
SADS-CoV	F ^1^	CTCGGCTTACTCTAAACCC	26,447–26,465	148
	R ^2^	CATCCACCATCTCAACCTC	26,577–26,595	
	P ^3^	FAM-AGTGTTCACTCCGGCACCTC-BHQ1	26,516–26,535	
PEDV	F	GAACCTAACACACCTCCT	26,779–26,796	147
	R	CACGACCCTGGTTATTTC	26,909–26,926	
	P	HEX-CAGAGGCAATAACCAGTCCCGT-BHQ1	26,868–26,889	
PDCoV	F	GTACATGGAGGTGCATTC	23,390–23,407	122
	R	GTGGCGGATTTCTAACTG	23,495–23,512	
	P	CY5-GGCTCCAATTCTCACACCAGTC-BHQ3	23,421–23,442	
TGEV	F	GCAGATGAGGTTGTTGCT	20,908–20,925	116
	R	GCCAGCGACTTCTAATGTTG	21,005–21,024	
	P	CY5.5-TGGTCTGGCACTGTCACATTTGGT-BHQ3	20,968–20,991	

^1^ Forword primer; ^2^ reverse primer; ^3^ probe.

## Data Availability

No datasets were generated or analyzed during the current study.

## Supplementary Materials

The following supporting information can be downloaded at: https://www.mdpi.com/article/10.3390/ijms26178731/s1.

## References

[1] Lai M., Cavanagh D. (1997). The Molecular Biology of Coronaviruses. Advances in Virus Research.

[2] Su S., Wong G., Shi W., Liu J., Lai A.C.K., Zhou J., Liu W., Bi Y., Gao G.F. (2016). Epidemiology, Genetic Recombination, and Pathogenesis of Coronaviruses. Trends Microbiol..

[3] Weiss S., Navas-Martín S. (2005). Coronavirus Pathogenesis and the Emerging Pathogen Severe Acute Respiratory Syndrome Coronavirus. Microbiol. Mol. Biol. Rev..

[4] Fang P., Fang L., Hong Y., Liu X., Dong N., Ma P., Bi J., Wang D., Xiao S. (2017). Discovery of a novel accessory protein NS7a encoded by porcine deltacoronavirus. J. Gen. Virol..

[5] Fu X., Fang B., Liu Y., Cai M., Jun J., Ma J., Bu D., Wang L., Zhou P., Wang H. (2018). Newly emerged porcine enteric alphacoronavirus in southern China: Identification, origin and evolutionary history analysis. Infect. Genet. Evol..

[6] Gong L., Li J., Zhou Q., Xu Z., Chen L., Zhang Y., Xue C., Wen Z., Cao Y. (2017). A New Bat-HKU2–like Coronavirus in Swine, China, 2017. Emerg. Infect. Dis..

[7] Pan Y., Tian X., Qin P., Wang B., Zhao P., Yang Y.-L., Wang L., Wang D., Song Y., Zhang X. (2017). Discovery of a novel swine enteric alphacoronavirus (SeACoV) in southern China. Vet. Microbiol..

[8] Lin C.-M., Saif L.J., Marthaler D., Wang Q. (2016). Evolution, antigenicity and pathogenicity of global porcine epidemic diarrhea virus strains. Virus Res..

[9] Lee S., Lee C. (2018). Genomic and antigenic characterization of porcine epidemic diarrhoea virus strains isolated from South Korea, 2017. Transbound. Emerg. Dis..

[10] Cui J., Li F., Shi Z.-L. (2019). Origin and evolution of pathogenic coronaviruses. Nat. Rev. Microbiol..

[11] Randall R., Goodbourn S. (2008). Interferons and viruses: An interplay between induction, signalling, antiviral responses and virus countermeasures. J. Gen. Virol..

[12] Shan X., Li R., Ma X., Qiu G., Xiang Y., Zhang X., Wu D., Wang L., Zhang J., Wang T. (2024). Epidemiology, pathogenesis, immune evasion mechanism and vaccine development of porcine Deltacoronavirus. Funct. Integr. Genom..

[13] Duan C., Wang J., Liu Y., Zhang J., Si J., Hao Z., Wang J. (2021). Antiviral effects of ergosterol peroxide in a pig model of porcine deltacoronavirus (PDCoV) infection involves modulation of apoptosis and tight junction in the small intestine. Vet. Res..

[14] Laude H., Rasschaert D., Delmas B., Godet M., Gelfi J., Charley B. (1990). Molecular biology of transmissible gastroenteritis virus. Vet. Microbiol..

[15] Wong A.C.P., Li X., Lau S.K.P., Woo P.C.Y. (2019). Global Epidemiology of Bat Coronaviruses. Viruses.

[16] Peng J.-Y., Shin D.-L., Li G., Wu N.-H., Herrler G. (2021). Time-dependent viral interference between influenza virus and coronavirus in the infection of differentiated porcine airway epithelial cells. Virulence.

[17] Zhou H., Shi K., Long F., Zhao K., Feng S., Yin Y., Xiong C., Qu S., Lu W., Li Z. (2022). A Quadruplex qRT-PCR for Differential Detection of Four Porcine Enteric Coronaviruses. Vet. Sci..

[18] Zhou P., Fan H., Lan T., Yang X.-L., Shi W.-F., Zhang W., Zhu Y., Zhang Y.-W., Xie Q.-M., Mani S. (2018). Fatal swine acute diarrhoea syndrome caused by an HKU2-related coronavirus of bat origin. Nature.

[19] Wang Q., Vlasova A.N., Kenney S.P., Saif L.J. (2019). Emerging and re-emerging coronaviruses in pigs. Curr. Opin. Virol..

[20] Wu Y., Li W., Zhou Q., Li Q., Xu Z., Shen H., Chen F. (2019). Characterization and pathogenicity of Vero cell-attenuated porcine epidemic diarrhea virus CT strain. Virol. J..

[21] Liu Y., Chen Y., Wang N., Qin H., Zhang L., Zhang S.-m. (2023). The global prevalence of parasites in non-biting flies as vectors: A systematic review and meta-analysis. Parasites Vectors.

[22] Antas M., Woźniakowski G. (2019). Current Status of Porcine Epidemic Diarrhoea (PED) in European Pigs. J. Vet. Res..

[23] Takahashi K., Okada K., Ohshima K.-I. (1983). An outbreak of swine diarrhea of a new-type associated with coronavirus-like particles in Japan. Jpn. J. Vet. Sci..

[24] Mole B. (2013). Deadly pig virus slips through US borders. Nature.

[25] Stevenson G.W., Hoang H., Schwartz K.J., Burrough E.R., Sun D., Madson D., Cooper V.L., Pillatzki A., Gauger P., Schmitt B.J. (2013). Emergence of Porcine epidemic diarrhea virus in the United States: Clinical signs, lesions, and viral genomic sequences. J. Vet. Diagn. Investig..

[26] Hu X., Li N., Tian Z., Yin X., Qu L., Qu J. (2015). Molecular characterization and phylogenetic analysis of transmissible gastroenteritis virus HX strain isolated from China. BMC Vet. Res..

[27] Jarvis M.C., Lam H.C., Rovira A., Marthaler D.G. (2016). Complete Genome Sequence of Porcine Epidemic Diarrhea Virus Strain COL/Cundinamarca/2014 from Colombia. Genome Announc..

[28] Woo P., Lau S., Lam C.S.F., Lau C., Tsang A.K.L., Lau J.H.N., Bai R., Teng J.L., Tsang C.C.C., Wang M. (2012). Discovery of Seven Novel Mammalian and Avian Coronaviruses in the Genus Deltacoronavirus Supports Bat Coronaviruses as the Gene Source of Alphacoronavirus and Betacoronavirus and Avian Coronaviruses as the Gene Source of Gammacoronavirus and Deltacoronavirus. J. Virol..

[29] Wang L., Byrum B., Zhang Y. (2014). Porcine coronavirus HKU15 detected in 9 US states, 2014. Emerg. Infect. Dis..

[30] Wang L., Byrum B., Zhang Y. (2014). Detection and genetic characterization of deltacoronavirus in pigs, Ohio, USA, 2014. Emerg. Infect. Dis..

[31] Jung K., Hu H., Saif L. (2017). Calves are susceptible to infection with the newly emerged porcine deltacoronavirus, but not with the swine enteric alphacoronavirus, porcine epidemic diarrhea virus. Arch. Virol..

[32] Zhang H., Ding Q., Yuan J., Han F., Wei Z., Hu H. (2022). Susceptibility to mice and potential evolutionary characteristics of porcine deltacoronavirus. J. Med. Virol..

[33] Piñeyro P.E., Lozada M.I., Alarcón L.V., Sanguinetti R., Cappuccio J.A., Pérez E.M., Vannucci F., Armocida A., Madson D.M., Perfumo C.J. (2018). First retrospective studies with etiological confirmation of porcine transmissible gastroenteritis virus infection in Argentina. BMC Vet. Res..

[34] Li Y., Niu J., Zhou X., Chu P., Zhang K., Gou H., Yang D.-X., Zhang J.-F., Li C.-l., Liao M. (2023). Development of a multiplex qRT-PCR assay for the detection of porcine epidemic diarrhea virus, porcine transmissible gastroenteritis virus and porcine Deltacoronavirus. Front. Vet. Sci..

[35] Chen Y., Zhang Y., Wang X., Zhou J., Ma L., Li J., Yang L., Ouyang H., Yuan H., Pang D. (2023). Transmissible Gastroenteritis Virus: An Update Review and Perspective. Viruses.

[36] Flores-Contreras E.A., Carrasco-González J.A., Linhares D.C.L., Corzo C.A., Campos-Villalobos J.I., Henao-Díaz A., Melchor-Martínez E.M., Iqbal H.M.N., González-González R.B., Parra-Saldívar R. (2023). Emergent Molecular Techniques Applied to the Detection of Porcine Viruses. Vet. Sci..

[37] Niu J.W., Li J.H., Guan J.L., Deng K.H., Wang X.W., Li G., Zhou X., Xu M.S., Chen R.A., Zhai S.L. (2022). Development of a multiplex RT-PCR method for the detection of four porcine enteric coronaviruses. Front. Vet. Sci..

[38] Pan Z., Lu J., Wang N., He W.T., Zhang L., Zhao W., Su S. (2020). Development of a TaqMan-probe-based multiplex real-time PCR for the simultaneous detection of emerging and reemerging swine coronaviruses. Virulence.

[39] Zhu J.H., Rawal G., Aljets E., Yim-Im W., Yang Y.L., Huang Y.W., Krueger K., Gauger P., Main R., Zhang J. (2022). Development and clinical applications of a 5-plex real-time RT-PCR for swine enteric coronaviruses. Viruses.

[40] Liu Q., Wang H.-Y. (2021). Porcine enteric coronaviruses: An updated overview of the pathogenesis, prevalence, and diagnosis. Vet. Res. Commun..

[41] Li J.Q., Cheng J., Lan X., Li X.R., Li W., Yin X.P., Li B.Y., Yang B., Li Z.Y., Zhang Y. (2010). Complete genomic sequence of transmissible gastroenteritis virus TS and 3’ end sequence characterization following cell culture. Virol. Sin..

[42] Hu W., Yu Q., Liqi Z., Liu H., Zhao S., Qi G., He K., Qian Y. (2014). Complete genomic sequence of the coronavirus transmissible gastroenteritis virus SHXB isolated in China. Arch. Virol..

[43] Yuan D., Yan Z., Li M., Wang Y., Su M., Sun D. (2021). Isolation and Characterization of a Porcine Transmissible Gastroenteritis Coronavirus in Northeast China. Front. Vet. Sci..

[44] Sun J., Zhang Q., Zhang C., Liu Z., Zhang J. (2023). Epidemiology of porcine deltacoronavirus among Chinese pig populations in China: Systematic review and meta-analysis. Front. Vet. Sci..

[45] Liu C., Huang W., He X., Feng Z., Chen Q. (2024). Research Advances on Swine Acute Diarrhea Syndrome Coronavirus. Animals.

[46] Saeng-chuto K., Madapong A., Kaeoket K., Piñeyro P.E., Tantituvanont A., Nilubol D. (2022). Co-infection of porcine deltacoronavirus and porcine epidemic diarrhea virus induces early TRAF6-mediated NF-κB and IRF7 signaling pathways through TLRs. Sci. Rep..

[47] Shahrajabian M.H., Sun W. (2024). The Significance and Importance of dPCR, qPCR, and SYBR Green PCR Kit in the Detection of Numerous Diseases. Curr. Pharm. Des..

[48] Cherry J.J., Kobayashi D.T., Lynes M.M., Naryshkin N.N., Tiziano F.D., Zaworski P.G., Rubin L.L., Jarecki J. (2014). Assays for the identification and prioritization of drug candidates for spinal muscular atrophy. Assay Drug Dev. Technol..

[49] Kuypers J., Jerome K.R. (2017). Applications of Digital PCR for Clinical Microbiology. J. Clin. Microbiol..

[50] Hayden R.T., Gu Z., Ingersoll J., Abdul-Ali D., Shi L., Pounds S., Caliendo A.M. (2012). Comparison of Droplet Digital PCR to Real-Time PCR for Quantitative Detection of Cytomegalovirus. J. Clin. Microbiol..

