# Supplementary material for: Establishment of Multiplex Digital PCR Assay for Detection of Four Porcine Enteric Coronaviruses
Source: Int J Mol Sci. 2025 Sep 8;26(17):8731. doi: 10.3390/ijms26178731 (PMC12429093; doi:10.3390/ijms26178731)
Supplement: Supplementary file 1 [file ijms-26-08731-s001.zip › ijms-3799171-supplementary.pdf]

Supplementary Materials

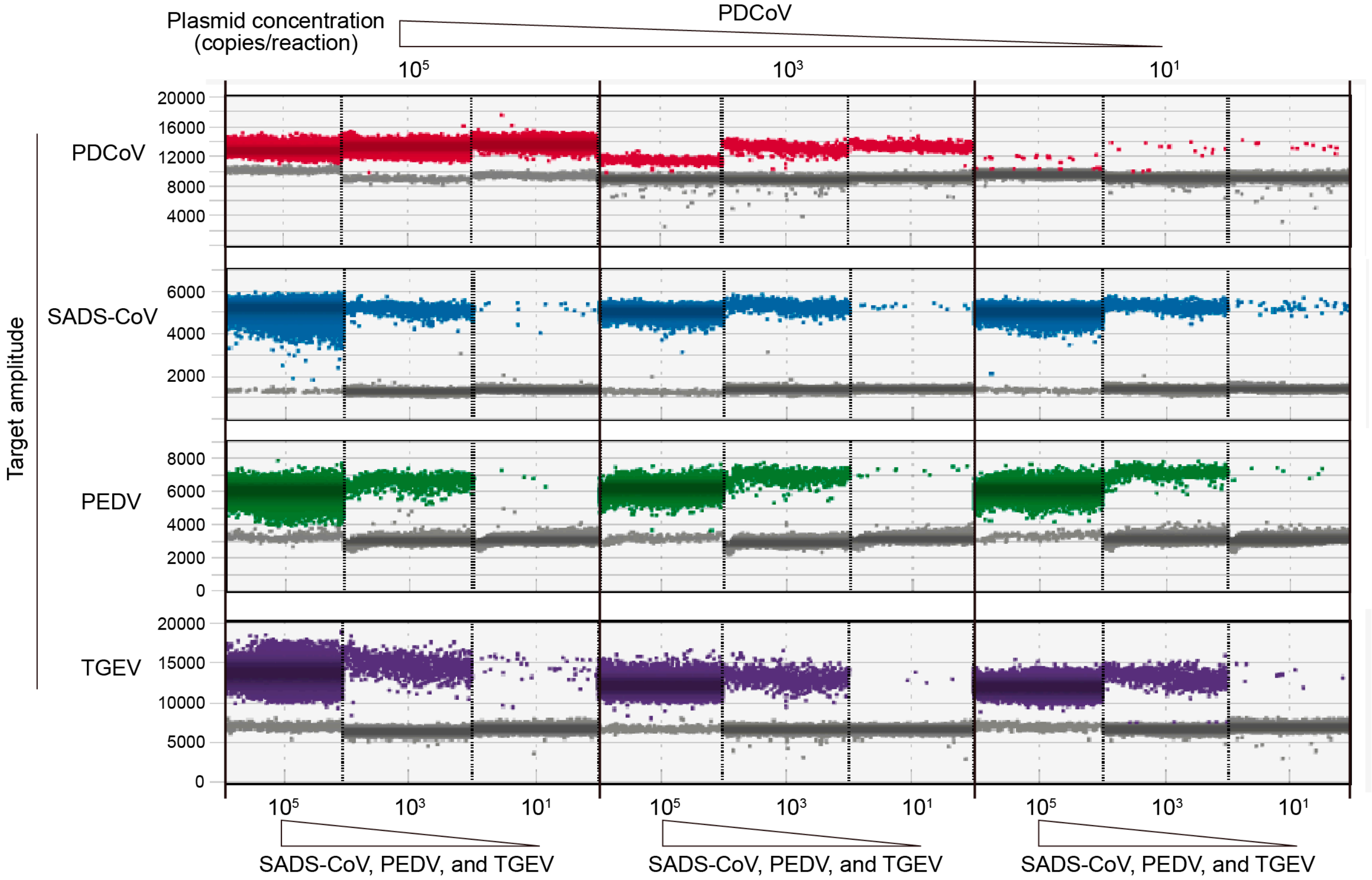

Figure S1. Interference resistance of multiple dPCR. Multiplex dPCR assays were performed after the arrangement and combination of PDCoV, SADS-CoV, PEDV and TGEV positive plasmids with concentration gradients from 10^5 to 10^1 copies/reaction.

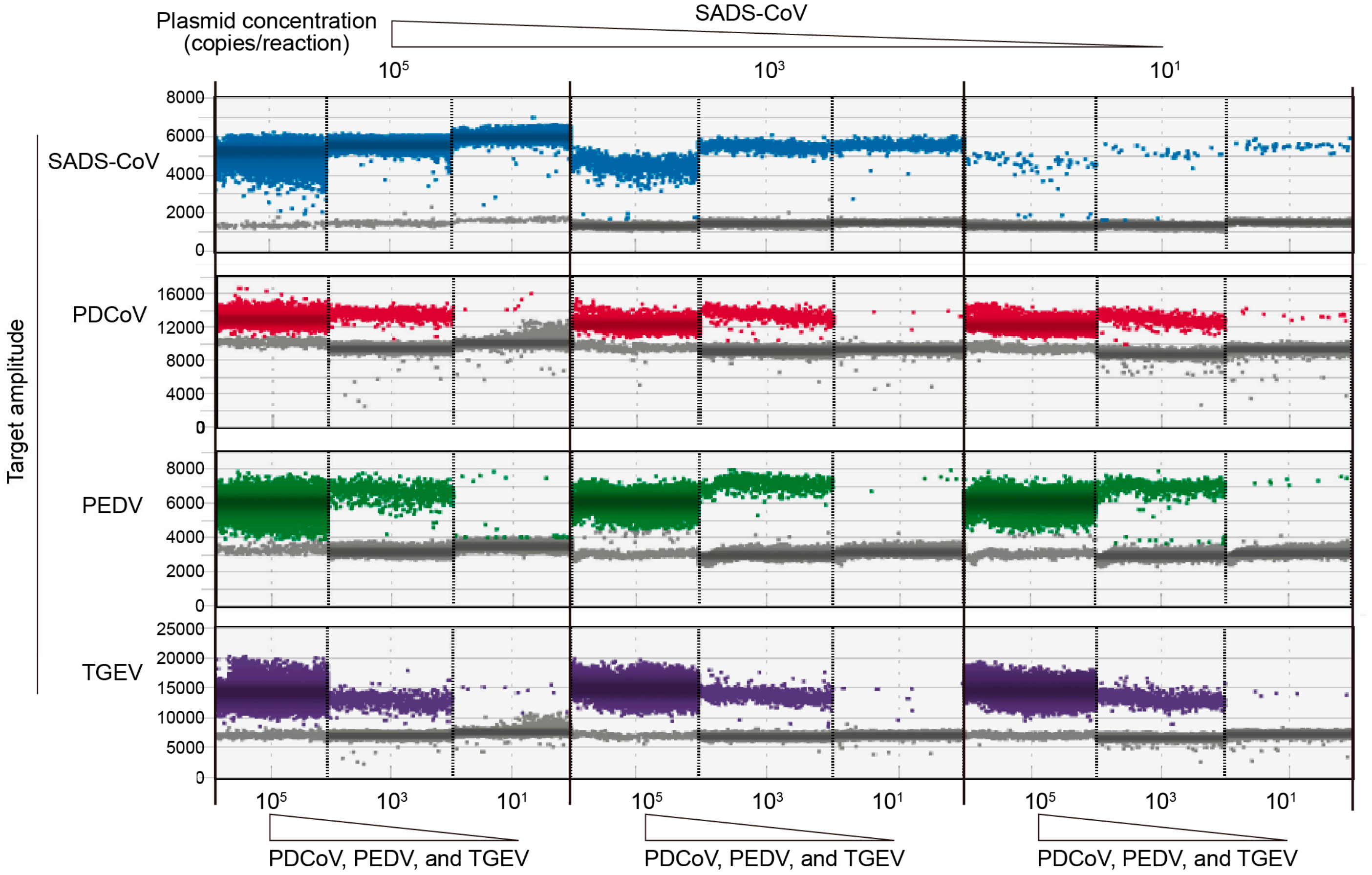

Figure S2. Interference resistance of multiple dPCR. Multiplex dPCR assays were performed after the arrangement and combination of SADS-CoV, PDCoV, PEDV and TGEV positive plasmids with concentration gradients from 10^5 to 10^1 copies/reaction.

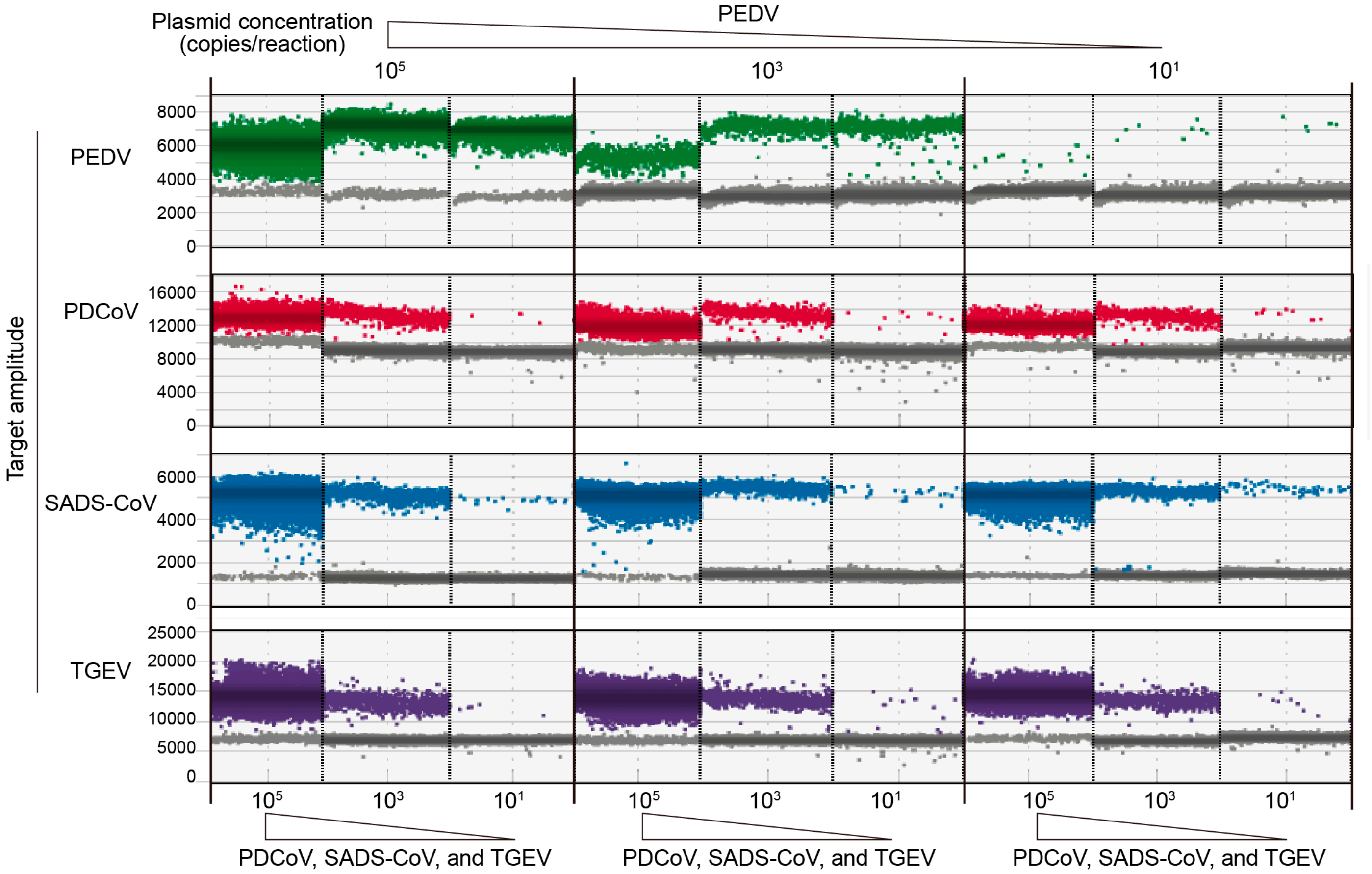

Figure S3. Interference resistance of multiple dPCR. Multiplex dPCR assays were performed after the arrangement and combination of PEDV, PDCoV, SADS-CoV, and TGEV positive plasmids with concentration gradients from 10^5 to 10^1 copies/reaction.

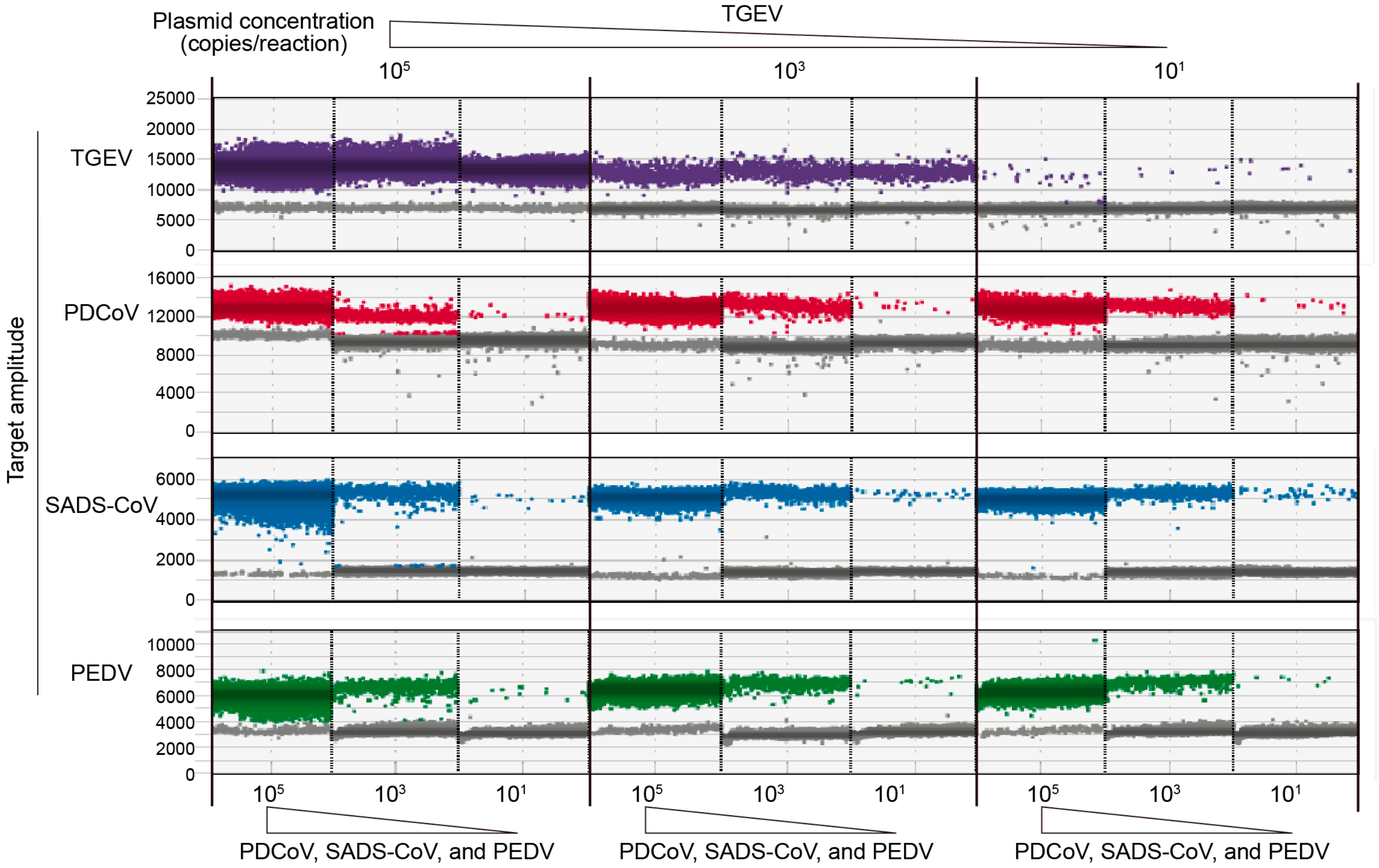

Figure S4. Interference resistance of multiple dPCR. Multiplex dPCR assays were performed after the arrangement and combination of TGEV, PDCoV, SADS-CoV, and PEDV positive plasmids with concentration gradients from 10^5 to 10^1 copies/reaction.
